# Serum Vitamin D Level and Rheumatoid Arthritis Disease Activity: Review and Meta-Analysis

**DOI:** 10.1371/journal.pone.0146351

**Published:** 2016-01-11

**Authors:** Jin Lin, Jian Liu, Michael L. Davies, Weiqian Chen

**Affiliations:** 1 Department of Rheumatology, the First Affiliated Hospital, College of Medicine, Zhejiang University, Hangzhou, Zhejiang Province, China; 2 Clinical Immunology Laboratory, Department of Microbiology and Immunology, Rosalind Franklin University of Medicine and Science, North Chicago, Illinois, United States of America; Harbin Medical University, CHINA

## Abstract

**Background:**

The evidence from epidemiological studies concerning the relationship between serum vitamin D concentrations and rheumatoid arthritis (RA) is inconsistent. This meta-analysis is aimed at determining the magnitude of the correlation between this common autoimmune disease and vitamin D, an important nutrient known to dampen adaptive immune responses.

**Methods:**

Through multiple search strategies, relevant literature was identified and evaluated for quality before May 16 2015. Data extracted from eligible studies was synthesized to calculate pooled correlation coefficient (r), mean difference (MD) and odds ratio (OR). The Venice criteria were applied to assess the credibility of the evidence for each statistically significant association.

**Results:**

A total of 24 reports involving 3489 patients were selected for analysis. RA patients had lower vitamin D levels than healthy controls (MD:-16.52 nmol/L, 95% confidence intervals [CI]:-18.85 to -14.19 nmol/L). There existed a negative relationship between serum 25-hydroxyvitamin D (25OHD) level and disease activity index, e.g. 25OHD *vs*. Disease Activity Score in 28 joints (DAS28): r = -0.13, 95% CI -0.16 to -0.09; 25OHD *vs*. C-reactive protein: r = -0.12, 95% CI -0.23 to -0.00. Additionally, latitude-stratified subgroup analysis yielded a relatively stronger negative correlation between 25OHD and DAS28 in low-latitude areas. This inverse relationship also appeared more significant in developing countries than in developed countries. No publication bias was detected.

**Conclusion:**

RA patients had lower vitamin D values than healthy controls. There was a negative association between serum vitamin D and RA disease activity. However, more strictly controlled studies are needed to validate these findings.

## Introduction

Rheumatoid arthritis (RA) is a chronic autoimmune disorder characterized by systemic features and joint involvement which affects 1% of the world’s adults [1]. It can lead to significant morbidity and mortality. Early diagnosis and proper treatment are crucial in decreasing the burden of this disease, but the pathogenesis and aetiology of RA remains unclear. Both genetic and nongenetic (e.g. environmental, infectious, hormonal) elements may be responsible for the prevalence of this disease [2]. Vitamin D might be one of the environmental factors relevant with RA [3]. There is a higher prevalence of osteoporosis in RA patients [4], and clinicians often supplement vitamin D together with calcium for this reason. Recent evidence demonstrates that vitamin D may correlate inversely with occurrence, development, disease activity and flare ups of RA [5–7]. The anti-inflammatory and immunomodulatory roles of vitamin D have gradually become apparent [8].

Vitamin D is a steroid hormone precursor that undergoes chemical conversion in the liver and kidney: the first reaction produces 25OHD, an objective indicator of vitamin D status, and the second produces the main bioactive form, 1,25-dihydroxyvitamin D (1,25(OH)_2_D) [9]. Vitamin D dampens the process of inflammation by influencing both innate and adaptive immune systems [10]. Dendritic cells and monocytes/macrophages belonging to the innate immune system express 1-alpha-hydroxylase to convert vitamin D to its active form 1,25(OH)_2_D and utilize it for autocrine and paracrine responses [10]. In the adaptive immune system, vitamin D downregulates expression of proteins involved in T helper type 1 (Th1) cell-driven autoimmunity, and participates in inhibition of antigen-presenting activity, antibody production, lymphocyte proliferation, dendritic cell differentiation and release of cytokines such as interleukin-2 (IL-2), IL-6, interferon-γ (IFN-γ) and tumor necrosis factor-α (TNF-α) [11–13]. In addition, vitamin D reinforces the development of monocytes into macrophages and then further influences their chemotaxis and cytokine expression [12]. Most autoimmune diseases, including inflammatory bowel disease, insulin-dependent diabetes mellitus, multiple sclerosis and rheumatoid arthritis, are Th1 cytokine-mediated disorders [14]. By dampening Th1 responses, vitamin D helps redirect the T cell response towards an immunosuppressive state.

There have been conflicting results regarding the correlation between RA and blood levels of 25OHD and 1,25(OH)_2_D [15–18]. These contradictions may be due to differences in factors such as design, study population, analytical methods, testing tools and sample size. A meta-analysis performed three years ago showed that vitamin D intake was inversely associated with risk of RA [19]. However, within the population of RA patients, that study was unable to analyze correlations between serum vitamin D level and RA disease activity, due to huge heterogeneity and a limited number of studies. During the past three years, more research has been published in this area. This allowed us to summarize the increased wealth of evidence now available and carry out a meta-analysis to explore serum vitamin D values in RA patients and ask whether vitamin D concentrations can be clearly correlated with disease activity of RA.

## Materials and Methods

### Search Strategy

Two reviewers independently screened potentially relevant studies via a systematic search of the literature covering the following computerized bibliographic databases: Pubmed, EBSCO, Web of Science and the Cochrane Library. Publication language was limited to English only and the last search was performed on May 16 2015. The subject terms and keywords used in our searches are as follows: “vitamin D”, "25OHD", "1,25(OH)_2_D", "rheumatoid arthritis" and "RA". Furthermore, reference lists of the recruited articles were searched manually to find additional work.

Inclusion criteria were as follows: (1) Predefined RA definition. (2) Data on serum vitamin D values and RA in an adult (>18 years) population. (3) For duplicate publications including overlapping data sets, only the latest or the most comprehensive study was included. (4) Observational studies of all designs. As for exclusion criteria, patients with conditions that might influence vitamin D levels were excluded, along with unhealthy controls that could introduce potential bias. Comments, review articles, case reports, letters, editorials, proceedings and unpublished articles (abstracts only) were also excluded.

### Data extraction

The following information was extracted independently by two reviewers (Jin Lin and Jian Liu) from each study: first author, publication year, country, diagnostic criteria, sample size, disease duration, age, sex, detection methods of vitamin D, vitamin D levels, and correlation coefficients between serum 25OHD (The major form of vitamin D considered in these studies) and disease activity index (Disease Activity Score in 28 joints [DAS28], serum C-reactive protein [CRP] and erythrocyte sedimentation rate [ESR]). One investigator (Jin Lin) compared extractions to ensure intercoder reliability and disagreement was coordinated by a third reviewer (Weiqian Chen) who did not take part in the original extraction.

Our study was approved by the ethics committee of the First Affiliated Hospital, College of Medicine, Zhejiang University.

### Study quality assessment

We did this meta-analysis in compliance with Meta-analysis Of Observational Studies in Epidemiology (MOOSE) guidelines [20] and Preferred Reporting Items for Systematic reviews and Meta-Analyses (PRISMA) guidelines [21]. Two observers independently evaluated the methodological quality of the included studies using the Newcastle—Ottawa Scale(NOS) [22] for case-control or cohort studies and the Agency for Healthcare Research and Quality (AHRQ) for cross-sectional studies [23]. The Venice criteria were considered to assess the credibility of the evidence for each statistically significant association in this manuscript [24].

### Statistical Analysis

In presenting the data on vitamin D values in RA patients and healthy controls, all incorporated studies used mean ± standard deviation (SD) except three [25–27] which used mean ± standard errors of means (SEM), thus we transformed SEM to SD to maintain consistency [28]. For evaluating vitamin D deficiency in RA patients and healthy controls, we calculated the pooled OR with the Mantel-Haenszel method. Vitamin D deficiency was defined as 25OHD<50 nmol/l according to published papers [29, 30]. When referring to the association between vitamin D and RA disease activity, Pearson correlation test was applied in most of the included articles, and for others we converted the Spearman correlation coefficients into Pearson correlation coefficients in order to minimize statistical errors [31]. Standard errors of correlation coefficients were calculated with Formula 1 and Formula 2. The 95% confidence intervals (CI) of each correlation coefficient was calculated using a Fisher’s z transformation (Formula 3) using the generic inverse variance method, after which all the values were transformed back to the original correlation coefficient metric (Formula 4) [32–34]. Formulas 1–4 are shown in S1 Fig.

Heterogeneity was assessed by using Cochran’s Q-statistic and inconsistency index (I^2^) test [35, 36]. An I^2^-value>50% or *P* value<0.05 suggested heterogeneity [37], and these data were calculated with DerSimonian & Laird random-effects models to accommodate diversity [38]. Otherwise, data were pooled with a fixed-effects model when there was no obvious heterogeneity [39].

We explored heterogeneity through a sequence of a priori secondary analyses. First, sensitivity analysis was conducted by excluding individual studies in turns for the purpose of discovering the potential influence of a single study on the final results. Second, Egger’s regression test was used to detect potential publication bias [39]. Third, we stratified the included studies according to measues of autoimmune disease activity (DAS28, ESR and CRP) to explicitly verify their relationship. Fourth, two subgroup analyses were made on the relationship between serum vitamin D levels and DAS28 based on latitude and economic status. Statistical difference between subgroups were calculated with the methods put forward by Snedecor, et al [40]. RevMan 5.3 software (Cochrane Library, Oxford, UK) and STATA 12.0 (Stata Corporation, TX, USA) were used for data acquisition and management.

### Data processing details

Two studies [27, 41] measured 25OHD in the same population during two seasons. As patients tend to suffer greater vitamin D deficiency and more severe arthritis symptoms in winter [42], the summer data was extracted to reduce false positive rate, which was also in agreement with most selected studies. Two other articles contained two datasets each, and each dataset was treated independently [42, 43].

Key confounders such as age, sex were accurately adjusted.

## Results

### Study characteristics

**Fig 1:**
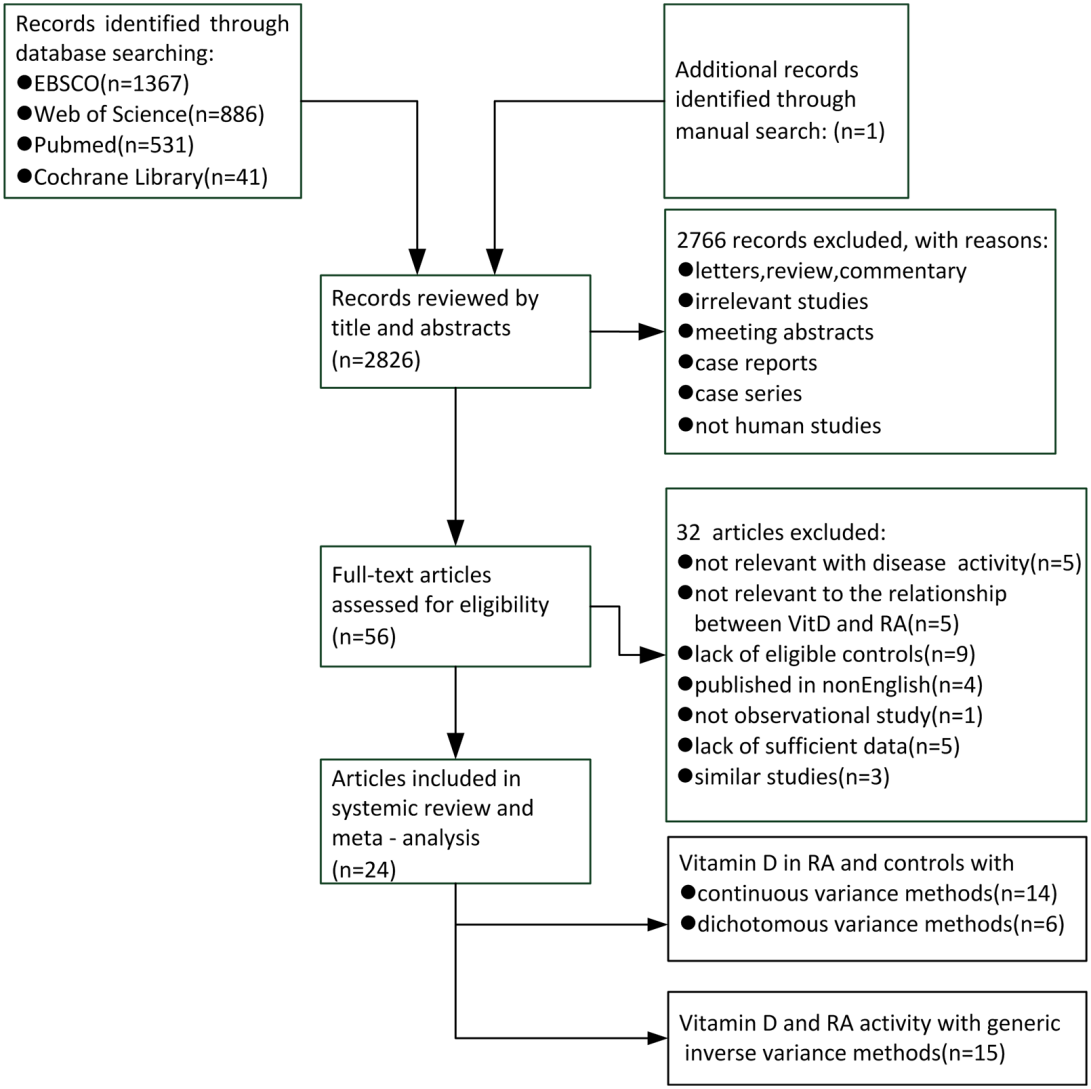
Flow diagram of the study selection process.

The study selection procedure is described in Fig 1. In the final analysis, two sets of studies with some results in common (Rossini [15] and Rossini [44]; Oelzner [45], Oelzner [46] and Oelzner [47]) were identified and the most comprehensive one in each set was singled out for our meta-analysis. Thus a total of 24 reports were enrolled. The eligible studies were published between 1998 and 2015 involving 3489 patients. Thirteen studies were performed in Asia, 5 in Europe, 4 in North America, 1 in South America and 1 in Africa. Most enrolled articles showed moderate to high quality on the basis of NOS scores. Fourteen studies were selected for analyzing differences of vitamin D values in RA patients and healthy controls. Six studies were employed to compare vitamin D deficiency rate in RA patients and healthy controls. Finally, we selected fifteen studies to investigate the relationship between serum vitamin D levels and severity of disease among RA patients. Table 1 shows the main features of the relevant studies.

**Table 1:** Characteristics of the included studies. r^D^: correlation between Vitamin D and DAS28; r^E^: correlation between Vitamin D and ESR; r^C^: correlation between Vitamin D and CRP; A: DAS28≥2.6; B: DAS28<2.6; NG: not given; RIA: radio-immunoassay; CLIA: chemiluminescent immunoassay; HPLC: high-performance liquid chromatography; ELISA: enzyme-linked immunosorbent assay; LCMS/MS: liquid chromatography tandem mass spectrometry; US: United States; ACR: American College of Rheumatology; EULAR: European League Against Rheumatism.

First author	Year	Country	VitD metabolite	Method	Diagnosis	Number of RA patients(Female)	Age (years)	Disease duration(years)	Correlation coefficient(r)
Kostoglou-Athanassiou [26]	2012	Greece	25OHD	RIA	1987ACR	44(NG)	NG	NG	r^D^ = -0.084, r^C^ = −0.115, r^E^ = -0.18
Haque [42]	2010	US	25OHD	NG	1987ACR	62(51)	57.6±12.9	11.6±12.3	A:r^D^ = -0.379, r^C^ = -0.134; B:r^D^ = -0.272, r^C^ = −0.402
Atwa [74]	2013	Saudi Arabia	25OHD	CLIA	1987ACR,2010ACR/EULAR	55(43)	45.60±12.41	4.93±3.11	r^D^ = -0.104, r^C^ = 0.051, r^E^ = -0.033
Yazmalar [41]	2013	Turkey	25OHD	HPLC	1987ACR	71(49)	45.30±10.55	NG	Summer:r^D^ = -0.099;Winter:r^D^ = 0.067
Sahebari [68]	2014	Iran	25OHD	ELISA	1987ACR	99 (NG)	43.94±14.31	5.9±5.6	r^D^ = 0.11, r^E^ = 0.74
Rossini [15]	2010	Italy	25OHD	ELISA	1987 ACR	1191(1014)	58.9 ± 11.1	11.5 ±8.7	r^D^ = -0.091
Baykal [75]	2013	Turkey	25OHD	ELISA	1987ACR	55(40)	45	NG	r^D^ = -0.15, r^E^ = -0.12, r^C^ = -0.14
Sabbagh [76]	2013	Canada	25OHD	NG	NG	39	NG	54.5±13	r^D^ = −0.43
Raczkiewicz [50]	2015	Poland	25OHD	CLIA	NG	97(86)	59.4 ± 12	8.1 ± 9	A: r^D^ = −0.26
Baker [77]	2012	Worldwide	25OHD	RIA	1987ACR	499 (416)	49.5±12.4	1.2	r^D^ = -0.08
Oelzner [45]	1998	Germany	25OHD,1,25(OH)_2_D	RIA	1987ARA	96(83)	54.7	12.2	25OHD:r^C^ = -0.14,1,25(OH)_2_D:r^C^ = −0.52
Gheita [48]	2014	Egypt	25OHD	CLIA	1987ACR,2010ACR/EULAR	63(49)	41.59±9.69	5.89±3.67	r^D^ = -0.34
Chen [78]	2014	China	25OHD	RIA	1987ACR	110(74)	59.48±11.41	6.51±6.82	r^D^ = –0.325
Braun-Moscovici [7]	2011	Israel	25OHD	RIA	1987ACR	85(NG)	55.8±14.1	9.9±8.5	r^D^ = -0.106
Matsumoto [79]	2015	Japan	25OHD	RIA	1987ACR	176(147)	61	10.2	r^D^ = -0.116
Azzeh [80]	2015	Saudi Arabia	25OHD	CLIA	2010ACR/EULAR	102(82)	50.09±10.98	NG	r^D^ = -0.277, r^E^ = -0.034
Cutolo [27]	2006	Italy/Estonia	25OHD	RIA	NG	117(NG)	58.5 ± 1.1	NG	r^D^ = - 0.57
Attar [51]	2012	Saudi Arabia	25OHD	LCMS/MS	1987ACR,2010ACR/EULAR	100(90)	47±13	4.7±5	r^D^ = -0.42
Sharma [81]	2014	India	25OHD	ELISA	1987ACR	80(NG)	40.97±12.52	NG	r^D^ = -0.604
Turhanoglu [82]	2011	Turkey	25OHD	ELISA	1987ACR	65(NG)	46.27±11.87	NG	r^D^ = -0.431, r^C^ = -0.276
Heidari [83]	2012	Iran	25OHD	ELISA	1987ACR	108(NG)	49.2 ± 13.1	NG	NG
Hong [49]	2014	China	25OHD	ELISA	1987ACR	130(95)	54± 14	6	NG
Brance [25]	2015	Argentina	25OHD	CLIA	2010ACR/EULAR	34(34)	52.2 ± 1.9	7.6 ± 1.4	NG
Hiraki [43]*	2014	US	25OHD	RIA	1987ACR	120(120)	63.8 ±8.2	NG	NG
Hiraki [43]*	2014	US	25OHD	RIA	1987ACR	46(46)	48.5±4.7	NG	NG

*This article included two nested case-control studies.

### Vitamin D values in RA patients

**Fig 2:**
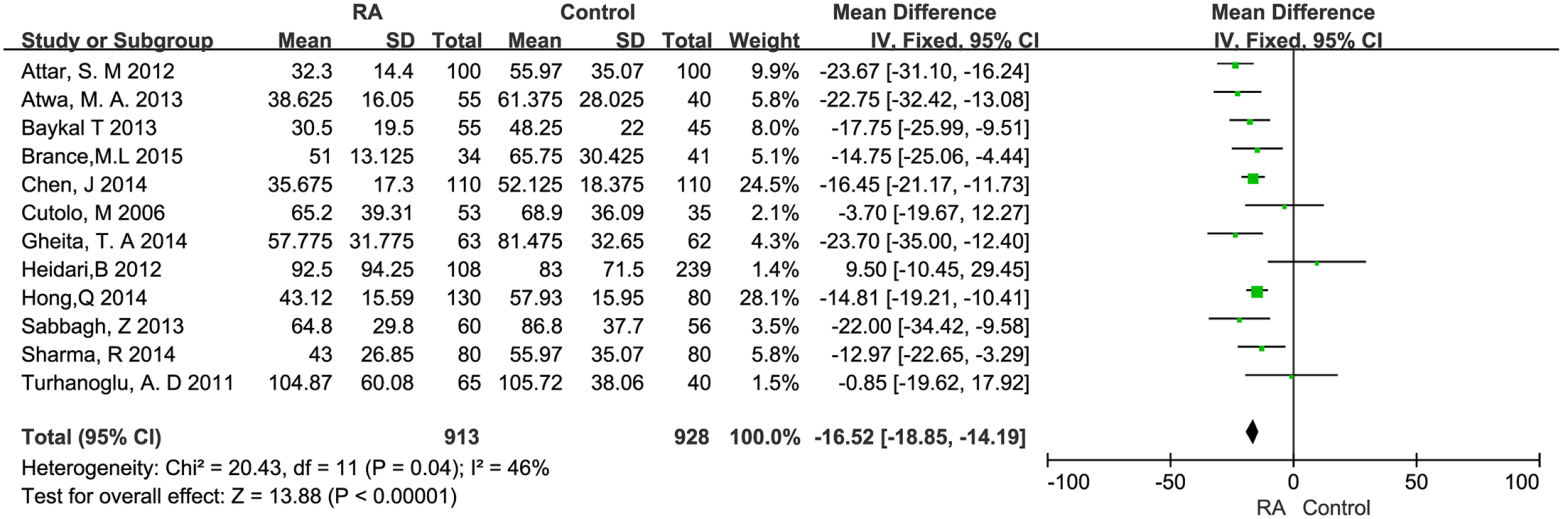
Forest plot of serum vitamin D values in RA patients and healthy controls using continuous variance methods.

2148 RA patients and 1991 healthy controls were selected for this quantitative synthesis. The average 25OHD level in RA patients was 13.21 nmol/L less than that in controls (mean difference [MD] = -13.21 nmol/L, 95% CI = -22.03 to -4.39 nmol/L) which exhibited notable heterogeneity (I^2^ = 95%, *P*<0.001). After sensitivity analysis, removing Rossini, et al and Kostoglou-Athanassiou, et al [15, 26] (the only outliers) remarkably reduced this heterogeneity with little influence on the outcome (MD = -16.52 nmol/L, 95% CI = -18.85 to -14.19 nmol/L, I^2^ = 46%, *P* = 0.04) (Fig 2). The Venice criteria grade was BBA, which resulted in a characterization as moderate evidence.

**Fig 3:**
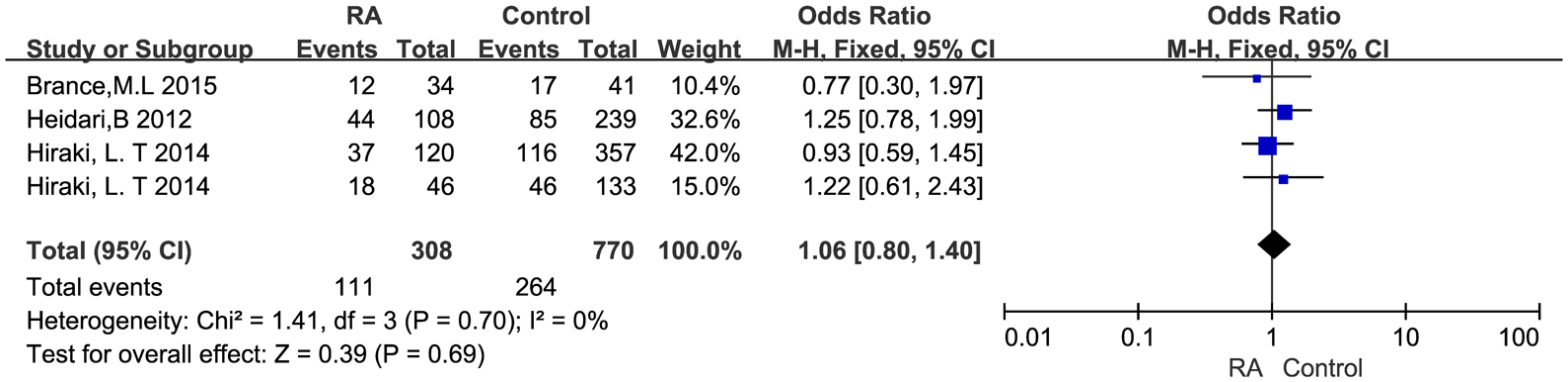
Forest plot of serum vitamin D values in RA patients and healthy controls using dichotomous variance methods.

We further selected six studies to compare vitamin D deficiency rate using dichotomous variance methods and did not find a significant difference between RA patients and healthy controls (OR = 1.49, 95% CI = 0.87 to 2.54, I^2^ = 88%, *P*<0.001). Three studies [15, 48, 49] were considered homogeneous after a sensitivity analysis and were excluded; comparing the remaining three studies showed a pooled OR = 1.06, 95% CI = 0.8 to 1.4, I^2^ = 0%, *P* = 0.7 (Fig 3).

### Relationship between vitamin D and RA activity

**Fig 4:**
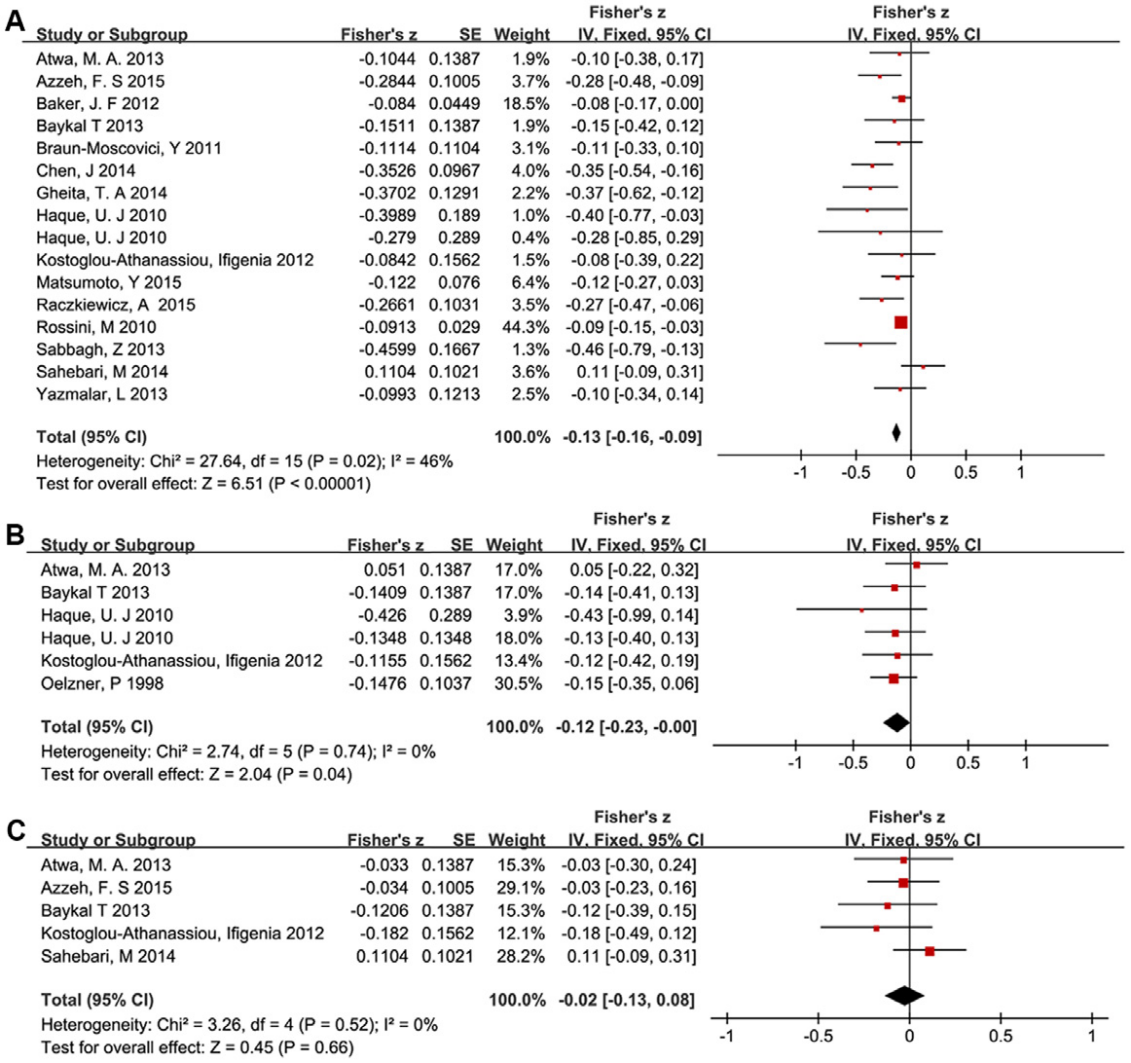
Forest plots of disease activity and serum vitamin D levels in RA patients. Summary of Fisher’s z tests with corresponding 95% confidence intervals for the relationship between: A) serum vitamin D levels and DAS28 scores; B) serum vitamin D levels and serum CRP levels; C) serum vitamin D levels and erythrocyte sedimentation rate.

We then specifically selected studies in which the association between serum vitamin D values and RA disease activity was presented with Pearson or Spearman correlation tests. The main outcome of this meta-analysis is summarized in Fig 4 and Table 2. All summary Fisher’s z scores were transformed back to r values as previously described. The pooled r for 25OHD compared to DAS28 (disease activity score in 28 joints) was -0.13 (95% CI -0.16 to -0.09, I^2^ = 46%, *P* = 0.02), producing a Venice criteria grade of ABA (Fig 4A). 25OHD was also inversely associated with serum CRP (r = -0.12, 95%CI -0.23 to -0.00, I^2^ = 0%, *P* = 0.74), producing a Venice criteria grade of BAA (Fig 4B). Both of these grades indicate moderately strong evidence for a correlation between increased 25OHD and reduced symptoms of RA disease. However, there was no significant correlation between 25OHD and erythrocyte sedimentation rate (r = -0.02, 95%CI -0.13 to 0.08, I^2^ = 0%, *P* = 0.52) (Fig 4C). Among these three analyses, the comparison of 25OHD to DAS28 had the most statistical power, as it incorporated 15 publications while the comparisons to CRP and ESR only incorporated 5 publications each.

**Table 2:** The main statistical results concerning vitamin D and RA disease activity. F: fixed-effects model; N: the relationship between vitamin D and RA was not statistically significant, and therefore we did not assess Venice Criteria grade.

	Studies	Number of patients	R(95%CI)	Heterogeneity test	Effects model	*P*_egger’s_	Venice Rating	Overall credibility
DAS28	15	2748	-0.13(-0.16,-0.09)	I^2^ = 46%, *P* = 0.02	F	0.321	ABA	Moderate evidence
CRP	5	312	-0.12(-0.23,-0.00)	I^2^ = 0%, *P* = 0.74	F	0.848	BAA	Moderate evidence
ESR	5	355	-0.02(-0.13,0.08)	I^2^ = 0%, *P* = 0.52	F	0.806	N	N

**Fig 5:**
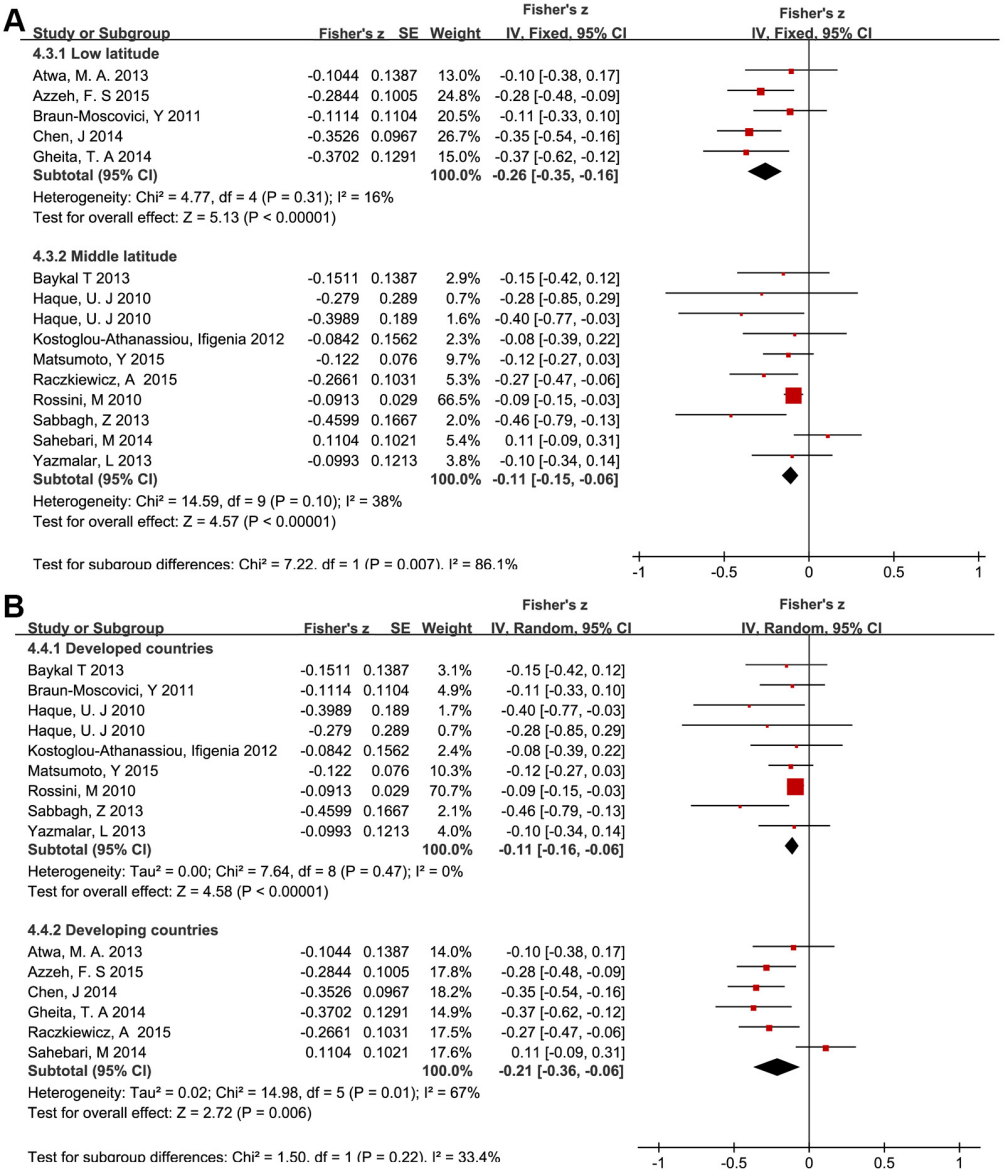
Forest plots of subgroup analysis focused on the relationship between serum vitamin D levels and disease activity scores in RA patients. Summary of Fisher’s z scores with corresponding 95% confidence intervals for the relationship between serum vitam D levels and DAS28 scores: A) stratified according to latitude; B) stratified according to economic status.

Finally, we conducted two subgroup analyses based on latitude and economic status of the locations where the studies were performed. The negative relation between vitamin D values and RA disease activity was more clearly displayed in low latitude areas as well as in developing countries. The results of the subgroup analyses and the Venice criteria grades are shown in Fig 5A and 5B and Table 3.

**Table 3:** Subgroup analysis results. F: fixed effects model; R: random effects model.

	Studies	Number of patients	R(95%CI)	Heterogeneity test	Effects model	Venice Rating	Overall credibility
Low latitude	5	415	-0.26(-0.35,-0.16)	I^2^ = 16%, *P* = 0.31	F	BAA	Moderate evidence
Middle latitude	9	1834	-0.11(-0.15,-0.06)	I^2^ = 38%, *P* = 0.10	F	AAA	Strong evidence
Developed countries	8	1723	-0.11(-0.16,-0.06)	I^2^ = 0%, *P* = 0.47	R	AAA	Strong evidence
Developing countries	6	526	-0.21(-0.36,-0.06)	I^2^ = 67%,P = 0.01	R	BCA	Weak evidence

### Publication bias and sensitivity analysis

Symmetrical funnel plots (S2 Fig) along with Egger’s regression test (Table 2) did not reveal any publication bias.

Further sensitivity analysis, by repeatedly reanalyzing the data after removing individual studies from the meta-analysis, demonstrated that no study was responsible for a disproportionate influence on the pooled estimates, indicating a statistically robust result of this analysis.

## Discussion

Through a comprehensive and quantitative meta-analysis, our results demonstrate that rheumatoid arthritis patients have lower vitamin D serum values than healthy controls, and that among RA patients there is a negative relationship between vitamin D serum values and RA disease activity. This meta-analysis follows two studies by independent groups that reported a significant inverse connection between serum 25OHD concentrations and DAS28 in patients with active RA (DAS28≥2.6) [42, 50]. However, Craig, et al [16] failed to discover a statistically significant association between 25OHD and DAS28 following multivariate adjustments in recent-onset rheumatoid arthritis patients. Cutolo, et al [27] found a remarkable negative correlation between 25OHD and DAS28 in summer only in southern European (Italian) patients and in winter in northern European (Estonian) patients, but no significant differences with respect to 25OHD values between Estonian and Italian RA patients and their controls. Attar, et al [51] discovered that 25OHD levels were only prominently lower in patients who poorly responded to therapy. To further investigate these links, we integrated a series of studies that included a considerable variety of disease durations, disease symptoms, and ethnicities, and we observed that the pooled correlations reached statistical significance. This is likely explained by the increased statistical power and resolution that meta-analysis makes possible via pooling the results of independent studies.

A systematic review published in 2010 [52] listed 7 studies evaluating vitamin D deficiency rate in RA patients compared with healthy controls. Two of these studies exhibited lower concentrations of vitamin D in RA, while the other five did not. However, this systematic review did not synthesize the data from each study to get an exact pooled estimate. Although our quantitative synthesis results did not find a significant statistical difference concerning the frequency of vitamin D deficiency (25OHD<50nmol/L) in RA patients and healthy controls, we did observe that 25OHD levels in RA patients are consistently lower than those in healthy control subjects. It can be inferred that low vitamin D levels may be associated with higher incidence of RA, as has already been mentioned by Song, et al [19].

Synovitis and pannus are the main pathological features of RA. CRP and ESR are non-specific inflammatory markers that have long been used for assessing systemic inflammation. DAS28 is a synthetically quantitative index which along with CRP and ESR can give a comprehensive picture of the inflammatory situations of RA patients [53]. In the current study, a considerable inverse association was observed between 25OHD levels and DAS28, as well as between 25OHD and CRP. However, the relationship between 25OHD and ESR was not as conclusive. This may be partly because CRP is a more direct measure of inflammation than ESR, and physiologically more sensitive to short-term changes, while ESR can continue to be elevated days after episodes of inflammation that have already resolved [53]. ESR is also influenced by many confounding factors such as temperature, age, gender, hematocrit and specimen handling technique; a normal population will have a greater variance in ESR values than CRP values, making it less likely that statistically significant ESR differences will be observed between groups [54]. Some experts have even proposed that CRP alone should be used as an alternative to ESR in the DAS28 calculation formula [55].

Subgroup analyses were implemented to further clarify the potential relationship between DAS28 and 25OHD. Strikingly, the latitude-stratified results demonstrated a stronger association in low-latitude areas. This may be explained by differences in duration of sun exposure and regional climates. For example, low-latitude areas tend to suffer more rain and the moist weather can give rise to greater arthritis pain which results in higher DAS28 scores. Subgroups classified by economic status similarly revealed a tighter relationship in developing countries. This may relate to differences in diet, hygiene, use of medications and vitamin supplements, or other factors; it has also been observed that RA is more common in wealthier countries but average RA activity is more severe in less developed countries [56, 57]. Increased correlation between vitamin D levels and RA in less wealthy countries may also stem from increased likelihood of exposure to environmental pollutants and infections, which could have synergistic effects exacerbating the effect of vitamin D deficiency [58].

The development of RA involves a diverse range of environmental and genetic factors. Indeed, studies a decade ago using murine models showed that vitamin D can prevent the incidence and progression of collagen-induced arthritis [59]. Other data have suggested that vitamin D receptor (VDR) agonists can suppress the severity of established collagen-induced arthritis [60]. VDR gene polymorphisms may influence vitamin D function, as well as its serum levels, and a recent meta-analysis displayed a significant contribution of *TaqI* and *FokI* VDR polymorphisms to RA risk [61]. Meanwhile, long-term use of vitamin D supplements can upregulate VDR signaling [62]. VDR can be found at high levels on immune system cells like dendritic cells, macrophages, and activated T and B lymphocytes, suggesting that the immunoregulatory action of vitamin D acts through many mechanisms [63]. Through binding VDR, vitamin D can suppress proinflammatory processes by inhibiting the enhanced activity of immune cells that participate in an adaptive autoimmune response. It appears to regulate immune homeostasis through reinforcing the innate response but impeding adaptive immunity. Vitamin D can also shift the balance from Th1 to Th2 and T regulatory cells [52], inhibiting the proliferation of Th1 cells that can exacerbate inflammation, generate bone loss and eventually lead to osteopenia and osteoporosis [64]. However, it has also been suggested that serum 25OHD levels may decrease in the acute-phase response, suggesting that low concentrations may be the result of high-grade systemic inflammation rather than a cause of inflammation [65]. Despite that, vitamin D concentrations were not affected when other phase reactants were reduced in rituximab- or adalimumab-treated RA patients [66, 67]. This cause-and-effect relationship may be elucidated by further research.

It is also worth noting that serum 25OHD concentrations did not correlate with disease activity in RA patients who were supplemented with physiological doses of vitamin D over a short period [7, 41, 68]. Therefore, investigating the optimal vitamin D cutoff point for predicting RA disease activity as well as for exerting its therapeutic functions would be clinically helpful.

The main active form of vitamin D, 1,25(OH)_2_D is responsible for most, if not all, of the immunoregulatory effects of vitamin D [69]. Oelzner, et al [45–47] reported a sequence of studies on the negative relevance between 1,25(OH)_2_D and RA disease activity. A randomised controlled trial operated by Gopinath, et al emphasized the pain relief action of 1,25(OH)_2_D in Disease Modifying Anti Rheumatic Drugs (DMARD)-naive RA [70]. However, most articles described in our manuscript did not consider 1,25(OH)_2_D in outcomes and we thus could not include it in our meta-analysis because of insufficient data.

As more and more research supports the idea of vitamin D as a regulatory factor in human immunity, correction of vitamin D deficiency may become a standard element of immunoregulation strategies in some autoimmune diseases. For example, Kim, et al [71] proposed that antirheumatic drugs combined with vitamin D should be recommended for RA. Vitamin D supplementation can prevent and treat osteoporosis at the same time, enhancing its possible effect on disease activity [72].

Several limitations exist in our analysis. First, the number of recruited patients in some studies was relatively small. However, stable results from sensitivity analysis, and the complete analysis of 24 different studies including a total of 3489 patients, strengthen our conclusion. Second, it could be suggested that due to variabilities in the way vitamin D was measured, pooling of the data was unsuitable. Nevertheless, the extracted information was consistent in terms of correlation coefficient, and our meta-analysis results exhibited no significant heterogeneity. Furthermore, these incorporated studies all came from institutions with high levels of expertise to conduct this type of experiment, such that high quality was very likely in most circumstances despite the variations. Third, despite our attempts to ensure inclusion of all relevant publications, and the lack of publication bias as illustrated by symmetrical funnel plots, we were unable to obtain unpublished or non-English research, and our pooled estimates might be overestimated if only significant or positive results have been reported. Fourth, meta-analyses of observational studies cannot be used for establishing a causative link. There has been extensive argument about whether researchers so far have adequately accounted for the potential confounding variables. However, even a randomized controlled trial is unlikely to conquer the many measurement challenges inherent to this topic. Similarly, the limitations of our analysis are also present in the original studies. For example, 25OHD levels can increase markedly with even transient exposure to ultraviolet B, and then persist for 2–3 weeks in the serum because of the long half-life of 25OHD compared to many serum biomarkers [73]. Hence the measurement of 25OHD with unawareness of disparities in sun exposure can introduce bias. Fifth, we are unable to use these results to suggest specific treatment strategies due to limited information.

## Conclusion

In conclusion, our meta-analysis indicates that serum vitamin D levels are lower in rheumatoid arthritis patients and are inversely associated with RA disease activity, particularly in low-latitude and developing nations. Further studies with larger sample sizes and more standardized, unbiased methods are required to elucidate a causal role of vitamin D in RA and thus to bring about new approaches for prevention and treatment of this disease.

## Supporting Information

S1 Fig Conversion formulas. (TIF)

S2 Fig Funnel plots of results. A. Funnel plot for vitamin D values in RA patients *vs*. healthy controls; B. Funnel plot for vitamin D deficiency in RA patients *vs*. healthy controls; C. Funnel plot for serum vitamin D *vs*. RA disease activity score (DAS28); D. Funnel plot for serum vitamin D *vs*. serum C-reactive protein; E. Funnel plot for serum vitamin D *vs*. erythrocyte sedimentation rate.
(TIF)

S1 PRISMA Checklist Checklist PRISMA. (DOC)
