# Supplementary figures and images for: Serum Vitamin D Level and Rheumatoid Arthritis Disease Activity: Review and Meta-Analysis
Source: PLoS One. 2016 Jan 11;11(1):e0146351. doi: 10.1371/journal.pone.0146351 (PMC4709104; doi:10.1371/journal.pone.0146351)

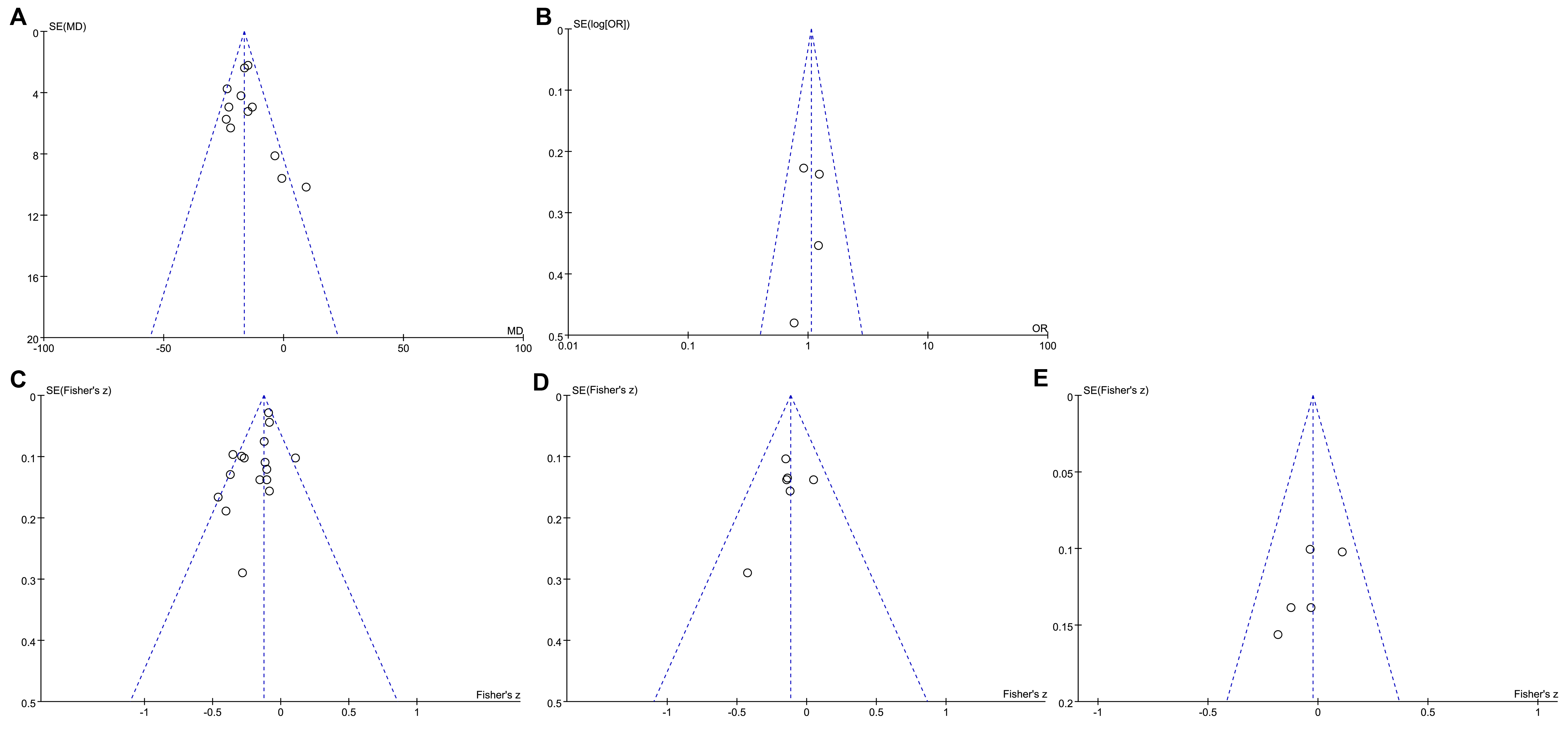

Supplement: S2 Fig — A. Funnel plot for vitamin D values in RA patients vs. healthy controls; B. Funnel plot for vitamin D deficiency in RA patients vs. healthy controls; C. Funnel plot for serum vitamin D vs. RA disease activity score (DAS28); D. Funnel plot for serum vitamin D vs. serum C-reactive protein; E. Funnel plot for serum vitamin D vs. erythrocyte sedimentation rate. (TIF) [file pone.0146351.s002.tif]
